# Factor structure and psychometric properties of the english version of the trier inventory for chronic stress (TICS-E)

**DOI:** 10.1186/s12874-018-0471-4

**Published:** 2018-02-06

**Authors:** Katja Petrowski, Sören Kliem, Michael Sadler, Alicia E. Meuret, Thomas Ritz, Elmar Brähler

**Affiliations:** 1 Witten/Herdecke University, Alfred-Herrhausen-Straße 50, 58448 Witten, Germany; 2 Criminological Research Institute of Lower Saxony, Lützerodestraße 9, 30161 Hannover, Germany; 3 eResearch Technology, 500 Rutherford Avenue, Boston, MA 02129 USA; 4 Department of Psychology, Southern Methodist University, 6116 N. Central Expressway, Suite 1160, Dallas, TX 75206 USA; 5 University Medical Center of the Johannes Gutenberg University Mainz, Langenbeckstraße 1, 55131 Mainz, Germany; 6 Department of Medical Psychology and Medical Sociology, University of Leipzig, Philipp-Rosenthal-Straße 55, 04103 Leipzig, Germany

**Keywords:** Chronic stress, Questionnaire, Confirmatory factor analysis, Measurement invariance, Stress assessment

## Abstract

**Background:**

Demands placed on individuals in occupational and social settings, as well as imbalances in personal traits and resources, can lead to chronic stress. The Trier Inventory for Chronic Stress (TICS) measures chronic stress while incorporating domain-specific aspects, and has been found to be a highly reliable and valid research tool. The aims of the present study were to confirm the German version TICS factorial structure in an English translation of the instrument (TICS-E) and to report its psychometric properties.

**Methods:**

A random route sample of healthy participants (*N* = 483) aged 18–30 years completed the TICS-E. The robust maximum likelihood estimation with a mean-adjusted chi-square test statistic was applied due to the sample’s significant deviation from the multivariate normal distribution. Goodness of fit, absolute model fit, and relative model fit were assessed by means of the root mean square error of approximation (RMSEA), the Comparative Fit Index (CFI) and the Tucker Lewis Index (TLI).

**Results:**

Reliability estimates (Cronbach’s α and adjusted split-half reliability) ranged from .84 to .92. Item-scale correlations ranged from .50 to .85. Measures of fit showed values of .052 for RMSEA (Cl = 0.50–.054) and .067 for SRMR for absolute model fit, and values of .846 (TLI) and .855 (CFI) for relative model-fit. Factor loadings ranged from .55 to .91.

**Conclusion:**

The psychometric properties and factor structure of the TICS-E are comparable to the German version of the TICS. The instrument therefore meets quality standards for an adequate measurement of chronic stress.

## Background

Current stress research has identified an increased risk for acute and chronic illnesses as well as impaired physical health as a result of chronic stress [4, 23]. Sleep disorders [34], the acute coronary syndrome [31], and chronic pain [13] may occur in individuals confronted with chronic stress. Consequently, there is the need for further analysis of the concept of chronic stress and investigative instruments that provide more insight into its considerable relevance to the physical and mental health of individuals of all ages [16].

The concept of chronic stress is based on the frequency, intensity and duration of stressors [14]. Chronic stress is defined as the repeated occurrence of various intense stressors with uncontrollable consequences over a sustained period of time in the absence of adaptive coping mechanisms [26, 30]. Chronic stress may also evolve from an individual’s ongoing lack of satisfaction with the fulfillment of his or her needs, e.g., the need for appreciation, social support, meaningful tasks, and diversification [33] or from *non-events*, e.g., desires for a marriage proposal or pregnancy, which fail to materialize [38, 39].

Social roles may provide boundaries to conceptualize the large array of chronic stressors such as interpersonal relationships, activities, obligations, and responsibilities which affect most people, particularly with respect to their workplace and their roles in partnerships [12, 24, 28].

According to the Systemic Requirement Resource Model of Health [3], an individual’s health may be promoted and preserved when the individual can depend on the availability of internal and external resources to satisfactorily deal with internal and external demands [36]. Internal demands are self-imposed based on an individual’s own values, needs, and personal strivings. Available external resources can be societal, ecological, personal or occupational. Psychological resources may include personality traits such as sense of coherence, self-efficacy, self-reliance, resilience, and the competence to apply resources appropriately.

The Stressor-Appraisal-Health Model identifies factors which can influence vulnerability to stressors [21]. Actual and perceived control over role stressors, emotional and instrumental social support, and the personality or disposition of the role incumbent all can be modifying factors (e.g., type A, optimism, negative affect). These factors are presumed to alter the role incumbent’s perceptions of role stressors and strengthen their ability to adapt successfully to role stressors [8]. In sum, individual health depends on the fulfillment of needs based on resources and modifier variables, and chronic stress may stem from the inability to cope with particular demands due to a lack of resources.

Theoretically, the impact of chronic stress is more relevant to the long-term development of illnesses than is the impact of acute stressors [22]. The human organism does not always habituate to chronic stressors lasting longer than 30 days, which may result in negative consequences for the immune system [17]. Experientially, there is no habituation; rather, higher levels of psychological distress symptoms are reported for stressors lasting up to one year [27].

Cohen et al. [10] provided an earlier overview of English-language assessment instruments for stress, e.g. the Perceived Stress Scale [9, 23]. However, chronic stress research mostly relies on life-event check-list measures. These scales provide a cumulative score based on either the number of events in a specific time frame or on the sum of the events’ weights based on ratings by judges [23]. This approach does not differentiate types of chronic stressors and their intensities. Other assessment instruments focus solely on the magnitude of stress in single areas or settings such as the workplace ([6]; [20]) or the parent-child system [1]. These research tools examine specific domains more closely, making them useful for specific research questions. However, for chronic stress that results from the repeated occurrence of different stressors [26, 30] a comprehensive instrument that covers various relevant aspects may prove more useful. The German Trier Inventory for Chronic Stress (TICS; [35]) is the first instrument that explicitly captures chronic psychosocial stress employing nine factors: Work Overload, Social Overload, Pressure to Perform, Work Discontent, Excessive Demands from Work, Lack of Social Recognition, Social Tensions, Social Isolation, and Chronic Worrying.

The authors of the TICS were inspired by Antonovsky [2] and other researchers who expanded the traditional model of risk factors by incorporating health protection aspects [11, 37]. They developed the Systemic Requirement - Resource Model of Health [3] in which individuals attempt to cope with external and internal demands by mobilizing external and internal resources. Thus, the model includes both the demands of the environment (private and work-related) as well as the demands that the person imposes upon the environment or him/herself. The resources implemented for these kinds of demands are in line with the multi-level model of physical and psychological resources (ecological, social, private and job–related, [3]). The nine factors of the TICS were developed in accordance with the Systemic Requirement - Resource Model of Health [3]. These factors can be grouped into High Demands referring to specific job conditions and social conditions, and Lack of Satisfaction of one’s needs due to unsatisfactory job conditions and social conditions [35]. As the TICS scales were developed based on the Systemic Requirement - Resource Model of Health [3], the authors postulated content validity as a logical consequence [35].

The factorial structure of the German TICS version, consisting of 57 items loading on nine factors was demonstrated in a sample of *N* = 604 adults (aged 16 to 70), who were randomly selected from telephone registers based on calculations of representative numbers of participants from the statistics of the German Federal Statistical Office for 1999 [35]. The authors of the TICS found a nine-factor model based on the item values and a two-factor model based on the sum scores of the nine scales [35]. Petrowski, Paul, Albani, & Brähler [29] subsequently replicated the psychometric properties and the factorial structure using a large representative German sample.

At present, there is no English-language questionnaire available to investigate chronic stress, that would fulfill the qualitative criteria of the German TICS [29]. A translated English-language version of the TICS might therefore be a valuable addition to the existing research tools for chronic stress. The aim of this research was to examine the quality criteria of the translated English-language version of the TICS with respect to its factorial structure and psychometric properties. In order to allow optimal comparison across language versions of the TICS we aimed at close correspondence with the German version of the TICS and did not plan to reduce the overall length of the instrument.

## Methods

### Sample

The data was collected at two college campuses in the Eastern and Southeastern region of the USA. Participants were undergraduate introductory psychology students who contributed in return for course credit. The final pooled sample of *N* = 483 participants with a mean age of *M* = 19.96 years (*SD* = 2.79, range = 18–30 years) included 75.6% female participants (*N* = 365). The participants were Caucasian (80.8%), African American (4.5%), Latino (1.2%), Asian American (5.2%), and other (6.4%). They received a data protection declaration that is in agreement with the Helsinki Declaration. The study was approved by the institutional review boards of the involved university institutions and all participants provided written informed consent.

### Instruments

The Trier Inventory for Chronic Stress (TICS) is a standardized German questionnaire that has been tested with respect to its factorial structure and psychometric properties, showing good to very good reliability [29]. Internal consistency (Cronbach’s Alpha, *α*) was good to very good with values ranging from .84 to .91 (mean of α = .87) [35]. Nine interrelated factors of chronic stress are assessed: Work Overload; Social Overload; Pressure to Perform; Work Discontent; Excessive Demands at Work; Lack of Social Recognition; Social Tensions; Social Isolation; Chronic Worrying. The nine factors were derived from 57 items rated on a five-point rating scale (0–4, labeled as: “never”, “rarely”, “sometimes”, “frequently”, “always”). Participants rated the occurrence/frequency of specific situations with a recall period of the previous three months. In addition, 12 items were summed for a screening scale.

The TICS was translated into English in accordance with the INTERNATIONAL TEST COMMISSION (ITC) Guidelines for Translating and Adapting Tests [19]. The items were translated from German to English by one bilingual expert and then back-translated to German by a second bilingual expert. Comparison and reconciliation of the original and back-translated items was carried out by a group of experts, followed by a second round of forward and back-translation. Sample items from each scale of the TICS-E are listed in Table 1.

**Table 1:** Sample TICS-E Items with scale allocation

Item number	Item	Scale
45	Arguments I get involved in frequently become lasting conflicts	Social Tensions
46	I feel that my performance is not recognized enough	Lack of Social Recognition
54	I feel overwhelmed by my tasks	Work Overload
55	In spite of the effort I make, I am unable to manage my tasks properly	Excessive Demands from Work
57	Sometimes I feel overburdened by my responsibilities toward others	Social Overload
37	I must meet responsibilities which I am adamantly opposed to	Work Discontent
25	Sometimes I am consumed by my worries	Chronic Worrying
29	Sometimes I lack the opportunity to articulate my concerns I have no opportunity to discuss things with others	Social Isolation
40	There are situations in which I find it difficult to be obliging	Pressure to Perform

### Statistical procedure

Internal consistency (Cronbach’s α) and split-half reliabilities were calculated for each of the TICS-E subscales. Skew and kurtosis were calculated on the item- and subscale-levels, and tests of normality (Shapiro-Wilk test) were performed. To determine item selectivity, the correlation of each item was computed with the remaining items on the respective scale (item-rest correlations). The main aim of this study was to examine the factorial validity of the TICS-E: A nine-factor model was examined using confirmatory factor analysis (CFA) with all items loading on their designated factor [29]. Due to significant deviations from the multivariate normal distribution, the robust maximum likelihood estimation (MLM) with a mean-adjusted chi-square test statistic (Satorra-Bentler χ2) was applied, which has been shown to be robust to violations of normality [7]. To evaluate the goodness of fit of the relevant model, four different (Satorra-Bentler.scaled) criteria were considered: the root mean square error of approximation (RMSEA.scaled), standardized root mean square residual (SRMR.scaled), and the 90% confidence interval to the absolute model fit. In addition, two criteria (the Comparative Fit-Index [CFI.scaled] and the Tucker Lewis Index [TLI.scaled]) were calculated to measure the relative model-fit compared to the “null” model. RMSEA values < .050 represent a “close fit”, RMSEA values between .050 and .080 represent a “reasonably close fit”, and RMSEA values > .100 represent an “unacceptable model” [5]. The SRMR with values less than 0.10, or of 0.08 (for a more conservative version; see [18]), are considered to represent a good fit. Regarding CFI and TLI, Hu and Bentler [18] suggested a CFI and TLI > .950 for a good model fit. Data analysis was carried out in R. The R package Latent Variable Analysis (lavaan); [32]) was used for the CFAs.

## Results

### Descriptive item analysis

Table 2 displays the means and standard deviations of the TICS-E subscales separately by gender, as well as the range of the selectivity values (r_*it*_) for the TICS-E items. Significant univariate non-normality was detected with the Shapiro-Wilk test for all subscales. Table 3 shows the moderate to high inter-correlations between the TICS-E subscales, TICS-E total score, and TICS-E-screening scale.

**Table 2:** TICS-E scale properties for the total sample

						Total sample *N* = 484				
			Reliabilities							
Scale	Items	Range^a^	Cronbach’s α	Split- half^b^	r_it_ range	*M*	*SD*	Skewness	Kurtosis	*W*
Work Overload	8	0–31 (0–32)	.89	.86	.59–.78	15.60	6.26	−0.20	0.38	0.99**
Social Overload	6	0–24 (0–24)	.87	.85	.62–.72	8.15	5.08	0.29	−0.33	0.97***
Pressure to Perform	9	0–35 (0–36)	.86	.84	.50–.68	17.64	6.23	−0.60	0.7	0.97***
Work Discontent	8	0–32 (0–32)	.86	.85	.56–.67	11.83	5.55	0.14	0.02	0.99***
Excessive Demands at Work	6	0–24 (0–24)	.85	.84	.52–.74	7.88	4.50	0.36	−0.12	0.98***
Lack of Social Recognition	4	0–16 (0–16)	.87	.87	.67–.80	5.36	3.14	0.32	0.05	0.97***
Social Tensions	6	0–24 (0–24)	.88	.86	.62–.75	5.81	4.26	0.57	0.05	0.95***
Social Isolation	6	0–24 (0–24)	.87	.86	.50–.77	8.85	4.90	0.35	0.11	0.98***
Chronic Worrying	4	0–16 (0–16)	.91	.91	.65–.85	6.80	4.17	0.26	−0.59	0.97***
Chronic Stress Screening Scale	12	0–46 (0–48)	.92	.82	.52–.78	19.87	9.43	0.08	−0.20	0.99***

*Note*. ^a^possible range of values in parentheses; ^b^Split-half reliabilities adjusted according to Spearman-Brown; ***r***_***it***_ *range* Range of item-scale correlations, *W* Test statistic related to the Shapiro-Wilk test

** = .05; *** = .001

**Table 3:** Intercorrelations of scales (upper matrix) and standardized factor covariances (lower matrix) of the English-language version of the TICS

Scale	Work Overload	Social Overload	Pressure to Perform	Work Discontent	Excessive Demands at Work	Lack of Social Recognition	Social Tensions	Social Isolation	Chronic Worrying	Chronic Stress Screening Scale
Work Overload	–	.54	.66	.56	.74	.58	.37	.49	.63	.83
Social Overload	.61	–	.60	.58	.56	.61	.54	.44	.47	.64
Pressure to Perform	.74	.69	–	.61	.54	.54	.42	.53	.55	.67
Work Discontent	.61	.66	.68	–	.7	.68	.58	.67	.55	.68
Excessive Demands at Work	.84	.64	.63	.81	–	.72	.55	.59	.65	.84
Lack of Social Recognition	.63	.70	.60	.78	.82	–	.59	.53	.58	.76
Social Tensions	.40	.62	.46	.67	.62	.67	–	.50	.38	.51
Social Isolation	.53	.48	.58	.76	.67	.57	.54	–	.57	.65
Chronic Worrying	.69	.51	.60	.57	.71	.60	.39	.59	–	.90
Chronic Stress Screening Scale										–

### Reliability estimates

As may be seen in Table 2, internal consistencies (Cronbach’s *α)* were good to very good and the adjusted split-half reliabilities ranged from .84 to .92. Table 3 presents item-scale correlations which ranged from r_it_ = .50 to .85. The item means were in the expected range.

### Confirmatory factor analysis

A CFA with items loading on the 9 relevant factors resulted in an adequate fit regarding RMSEA**.scaled** = .052 (CI = .050–.054) and SRMR**.scaled** = .067 and an unacceptable comparative fit according to the TLI**.scaled** = .846 and CFI**.scaled** = .855. However, the model fits are comparable in structure to the results obtained by Petrowski et al. [29] with the German version of the TICS (TLI = .855, CFI = .863, SRMR = .071, RMSEA = .051). Table 3 shows the estimated inter-factor correlations based on the results of the CFA. Factor-loadings are presented in Table 4, ranging from .55 to .91.

**Table 4:** Item-scale correlation and factor loadings for the items of the English version of the TICS

						9-factor model					
Item	M	SD	Skewness	Kurtosis	W	Factor	Factor loading	Standardized error variances	Factor	Factor loading	Standardized error variances
						*Work Overload*			High Demand		
01	2.20	0.96	−0.45	−0.23	0,89		.62	.62		.57	.68
04	1.74	1.04	0.17	−0.53	0.91		.62	.62		.57	.68
17	1.33	1.06	0.45	−0.50	0.89		.63	.60		.61	.63
27	2.16	1.07	−0.26	−0.46	0.91		.60	.64		.60	.64
38	1.80	1.05	−0.11	−0.62	0.91		.77	.41		.74	.45
44	2.18	1.05	−0.26	−0.35	0.91		.80	.36		.69	.52
50	1.90	1.06	0.02	−0.50	0.91		.79	.38		.68	.54
54	2.29	1.05	−0.31	−0.34	0.91		.83	.31		.72	.48
						*Social Overload*					
07	1.39	1.16	0.49	−0.63	0.89		.66	.56		.54	.71
19	1.57	1.10	0.27	−0.66	0.91		.78	.39		.68	.54
28	1.30	1.03	0.54	−0.21	0.88		.74	.45		.59	.65
39	1.43	1.12	0.40	−0.57	0.89		.77	.41		.63	.60
49	1.23	1.11	0.58	−0.46	0.87		.66	.56		.53	.72
57	1.24	1.04	0.47	−0.51	0.88		.72	.48		.61	.63
						*Pressure to Perform*					
08	1.49	0.99	0.16	−0.50	0.90		.55	.70		.55	.70
12	1.86	1.01	0.00	−0.42	0.91		.60	.64		.54	.71
14	1.54	0.97	0.14	−0.51	0.90		.55	.70		.55	.70
22	1.99	1.02	−0.21	−0.34	0.90		.73	.47		.65	.58
23	2.40	0.97	−0.55	0.28	0.88		.59	.65		.46	.79
30	1.68	1.00	0.00	−0.50	0.90		.58	.66		.52	.73
32	2.51	1.00	−0.61	0.22	0.88		.64	.59		.55	.70
40	2.14	1.07	−0.36	−0.36	0.91		.75	.44		.69	.52
43	1.49	0.99	0.16	−0.50	0.90		.71	.50		.66	.56
						*Work Discontent*			Lack of Satisfaction		
05	2.02	0.98	−0.19	−0.25	0.90		.57	.68		.50	.75
10	1.29	1.06	0.50	−0.40	0.88		.67	.55		.61	.63
13	2.07	0.86	−0.19	0.15	0.88		.60	.64		.55	.70
21	1.23	0.92	0.43	−0.08	0.87		.73	.47		.66	.56
37	1.18	0.99	0.49	−0.51	0.87		.69	.52		.67	.55
41	1.05	0.88	0.58	0.01	0.85		.69	.52		.62	.62
48	1.38	1.01	0.35	−0.36	0.89		.68	.54		.62	.62
53	1.61	1.07	0.12	−0.67	0.91		.67	.55		.64	.59
						*Excessive Demands at Work*					
03	1.46	0.96	0.35	−0.18	0.89		.70	.51		.62	.62
20	1.19	0.97	0.66	0.23	0.87		.69	.52		.64	.59
24	0.67	0.86	1.14	0.64	0.75		.57	.68		.55	.70
35	1.36	0.91	0.21	−0.31	0.88		.70	.51		.67	.55
47	1.80	1.16	0.13	−0.73	0.91		.75	.44		.70	.51
55	1.39	1.06	0.46	−0.46	0.89		.80	.36		.73	.47
						*Lack of Social Recognition*					
02	1.64	0.87	0.00	−0.22	0.88		.72	.48		.64	.59
18	1.13	0.90	0.54	0.01	0.86		.80	.36		.72	.48
31	1.26	0.92	0.40	−0.12	0.88		.86	.26		.72	.48
46	1.33	1.00	0.42	−0.25	0.89		.81	.34		.73	.47
						*Social Tensions*					
06	1.24	0.86	0.34	−0.30	0.87		.73	.47		.57	.68
15	1.06	0.89	0.50	−0.31	0.86		.72	.48		.54	.71
26	0.71	0.90	1.19	0.95	0.76		.67	.55		.50	.75
33	1.01	0.95	0.88	0.57	0.84		.72	.48		.56	.69
45	0.84	0.90	0.94	0.51	0.81		.82	.33		.60	.64
52	0.96	0.92	0.75	0.07	0.84		.76	.42		.54	.71
						*Social Isolation*					
11	1.34	1.04	0.51	−0.35	0.89		.74	.45		.57	.68
29	1.39	1.00	0.41	−0.25	0.89		.58	.66		.62	.62
34	1.45	1.06	0.22	−0.68	0.90		.80	.36		.63	.60
42	1.28	1.00	0.52	−0.19	0.88		.83	.31		.61	.63
51	1.68	1.03	0.07	−0.47	0.91		.72	.48		.55	.70
56	1.70	1.17	0.15	−0.75	0.91		.72	.48		.61	.63
						*Chronic Worrying*					
09	1.54	1.12	0.33	−0.63	0.90		.68	.54		.67	.55
16	1.85	1.20	0.08	−0.87	0.91		.89	.21		.64	.59
25	1.69	1.18	0.22	−0.78	0.91		.90	.19		.66	.56
36	1.72	1.21	0.25	−0.83	0.91		.91	.17		.67	.55

We also analyzed the aforementioned two factor model. This model resulted in an inadequate fit according to RMSEA.scaled = .079 (CI = .077–.081) and SRMR.scaled = 0.080 and an unacceptable comparative fit according to the TLI.scaled = .644 and CFI.scaled = .080. Overall the two-factor solution did not meet any of the common tests of model fit. Factor-loadings are also presented in Table 4, ranging from .46 to .74.

## Discussion

Our findings indicate that the translated version of the German TICS shows good reliability and an adequate fit for the nine-factorial model based on CFA.

### Confirmatory factor analysis (CFA)

The 9-factor model was previously found to be superior to the 2-factor model of the TICS [29]. The 9-factor model in the present English version of the TICS (TICS-E) produced some fit indices of CFA comparable to the earlier replication study of the German version [29], specifically the RMSEA and SRMR of the TICS-E indicated an adequate model fit [15]. On the other hand, the TLI and CFI values of the TICS-E showed an unacceptable comparative fit according to the guidelines of Hu & Bentler [18]. These lower values align with the TLI and CFI results found for the German version of the TICS [29]. These mixed results might be accounted for by the large number of nine different factors [29], as well as the inability of large questionnaire data to fully meet the recommendations for goodness-of-fit suggested by Hu & Bentler [18] and Marsh, Hau, & Wen [25]. Some factor loadings (Table 3) were in the area of .5 but most were greater than .7. Thus, all items were strongly related to their associated latent constructs. [15]. For practical use, both the nine individual subscales and the total score can be used. The inter-correlations between subscales ranged between .37 to .90, which was higher than for the original results with the German TICS version *r* = .15 to .63 [35]. However, the inter-correlations were in the same range as in the replication study of the German TICS (ranging from *r* = .30 to .77; [29]). The factorial structure of the TICS-E could also be replicated with a model fit similar to the original German version.

### Reliability and descriptive scale analysis

The TICS-E showed good to very good internal consistency and reliability (Cronbach’s *α* = .84–.92; split-half reliabilities *r* = .82–.91). The relevant values aligned well with the results as published in the original TICS manual by Schulz et al. [35] (Cronbach‘s *α* = .84 to.91). Although the sample composition differed from that described by Petrowski et al. [29] with respect to mean age and gender, reliability estimates were only marginally different, with Cronbach’s α (*α* = .81–.91) and split-half reliabilities (*r* = .81–.90) being slightly higher for the TICS-E [29]. The item scale correlations ranged from *r*_*it*_ = 0.5 to 0.85 and were therefore higher than for the German TICS version (item scale correlations *r*_*it*_ = .48 to .80). The item means were similar to the data from the representative German sample [29]. However, the scale scores Work Overload and Pressure to Perform were slightly higher and Social Isolation and Chronic Worrying were slightly lower than in the German reference samples. Overall, the TICS-E showed good psychometric properties.

### Limitations

The sample of the present study does not allow inferences about the US population as a whole because it was drawn from the undergraduate student population, with a low mean age of 19.96 years (SD = 2.79), consisting mostly of female participants (75.6%). The socioeconomic background and work experiences of these students are very homogeneous and the variance of chronic work or family stress is limited. In addition, it is likely that these psychology students have experience with answering questionnaires. It is possible that this also affected the evaluation of the psychometric properties of the questionnaire. This limitation is shared with previous studies using questionnaires. Additional limitations regarding questionnaire validity are posed by the use of a rating scale with only five response categories and the suboptimal model fit in the CFA.

### Future research indications

Future studies could test the stability of the chronic stress construct by investigating test-retest reliability and external relationships (criterion validity). In addition, evaluation of the TICS-E in a broader, population-based sample is needed. The best possible comparison would be between representative samples of native speakers of both languages. Additionally, research should explore the convergent validity of the TICS-E by examining associations with other chronic stress measures. Study designs with multiple assessment points would provide the opportunity to compare the stability of the questionnaire’s factor structure and determine possible cohort effects. The stress concepts of the questionnaires are based on research conducted in the United States [2, 11]. The English and German versions of the TICS might also be used to study chronic stress concepts across cultures (German and American).

## Conclusion

We conclude that the psychometric properties and factor structure of the TICS-E are largely comparable to the German version of the TICS in previous studies. The English version meets the relevant requirements of good psychometric properties and factorial validity and is therefore a promising instrument for measuring chronic stress.

## Abbreviation

CFA: Confirmatory Factor Analysis
CFI: Comparative Fit Index
ITC: International Test Commission
MLM: Maximum Likelihood Estimation (MLM)
RMSEA: Root mean square error of approximation
SRMR: Standardized root mean square residual
TICS: Trier Inventory for Chronic Stress
TICS-E: English version of the Trier Inventory for Chronic Stress
TLI: Tucker Lewis Index
